# Effect of a story-based, animated video to reduce added sugar consumption: A web-based randomized controlled trial

**DOI:** 10.7189/jogh.11.04064

**Published:** 2021-10-30

**Authors:** Alain Vandormael, Violetta Hachaturyan, Maya Adam, Caterina Favaretti, Jennifer Gates, Till Bärnighausen

**Affiliations:** 1 Heidelberg Institute of Global Health, Heidelberg University, Heidelberg, Germany; 2 Department of Pediatrics, Stanford University School of Medicine, Stanford, California, USA; 3 Icahn School of Medicine, Mount Sinai, New York, New York, USA; 4 Africa Health Research Institute (AHRI), Durban, South Africa.; 5 Department of Global Health and Population, Harvard T.H. Chan School of Public Health, Boston, Massachusetts, USA

## Abstract

**Background:**

Short and animated story-based (SAS) videos, which can be rapidly distributed through social media channels, are a novel and promising strategy for promoting health behaviors. In this study, we evaluate the effectiveness of a SAS video intervention to reduce the consumption of added sugars.

**Methods:**

In December 2020, we randomized 4159 English-speaking participants from the United Kingdom (1:1:1) to a sugar intervention video, a content placebo video about sunscreen use (no sugar message), or a placebo video about earthquakes (no health or sugar message). We nested six list experiments in each arm and randomized participants (1:1) to a control list or a control list plus an item about consuming added sugars. The primary end-points were mean differences (on a scale of 0-100) in behavioral intent and direct restoration of freedom to consume added sugars.

**Results:**

Participants (N = 4013) who watched the sugar video had significantly higher behavioral intent to cut their daily intake of added sugar (mean difference (md) = 16.7, 95% confidence interval (CI) = 1.5-31.8, *P* = 0.031), eat fresh fruit daily (md = 16.7, 95% CI = 0.5-32.9, *P* = 0.043), and check food labels for sugar content (md = 20.5, 95% CI = 2.6-38.5, *P* = 0.025) when compared with the sunscreen (content placebo) video. The sugar video did not arouse intent to restore freedom and consume added sugars when compared with the two placebo videos.

**Conclusions:**

Our SAS intervention video did not arouse reactance and increased short-term behavioral intent among participants to reduce their consumption of added sugars. SAS videos, which draw on best practices from the entertainment-education media, communication theory, and the animation industry, can be an effective strategy for delivering emotionally compelling narratives to promote health behavior change.

**Trial registration:**

German Clinical Trials Register: DRKS00022340

Short and animated story-based (SAS) videos are a novel and promising strategy for promoting health behaviors. This strategy draws from entertainment-education media [1,2], communication theory, and the animation industry [3] to create compelling, evidence-based health messages that virtually ‘go viral’ [4]. SAS videos can be developed and rapidly deployed on social media channels, avoiding the delays typically associated with sluggish public health campaigns [5-8]. Importantly, SAS videos can creatively sidestep social barriers and convey health messages that are accessible across languages, ages, cultural affiliations, and education levels [4,5]. Research has shown that packaging health recommendations in a relatable story can be more effective than traditional media approaches that frame health messages as informational arguments [9,10]. To gain further traction as a health communication tool, SAS videos must demonstrate their impact in reducing the knowledge translation gap and improving intent toward the targeted health behavior [11,12].

To be effective, SAS videos will have to overcome the same challenges faced by other traditional methods of health persuasion [13]. These methods often fail to achieve the desired effect [14], and in some cases, may arouse a motivation to reject a message, a phenomenon known as reactance [15]. The theory of reactance posits that individuals will seek to restore their freedom – after it has been eliminated or threatened with elimination – by performing the forbidden act [16]. In **Figure 1****,** we show a pathway through which a persuasive health message can arouse the direct restoration of freedom [14-17]. This conceptual model also shows how a persuasive message is mediated by the antecedents, components, and consequences of reactance to affect behavioral intent. A SAS intervention video should aim to attenuate direct restoration and increase intent toward the promoted health behavior.

**Figure 1:**
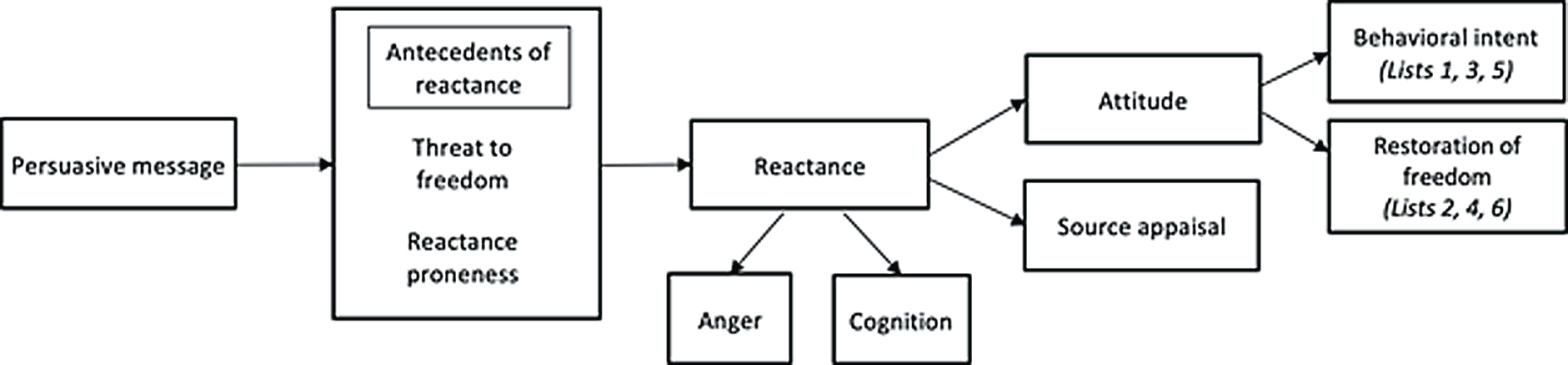
An Intertwined Process Cognitive-Affective Model adapted from Dillard & Shen [14] and Zhang [17]. In this model, there are two antecedents to reactance: strength of the threat to freedom and trait proneness to reactance. Reactance is conceptualized as a mediator between the antecedents of reactance and behavioral intent to undertake the promoted health activity. It is an intertwined process consisting of a cognitive and affective component, which is an experience of hostile, aggressive or angry feelings. Further, attitudinal and behavioral intentions are the consequences of reactance. The assessment of behavioral intentions can also help to measure direct restoration of freedom, which involves performing the forbidden behavior and restoring participant’s need for self-determination and control.

In this study, we investigate the effectiveness of a SAS video to reduce the consumption of added sugars [18]. The excessive intake of added sugars has increased worldwide and been associated with numerous adverse health outcomes, including obesity, type 2 diabetes, and cardiovascular disease [19]. Several interventions have used social media channels to distribute video-based messages about the harmful effects of added sugars. However, a recent systematic review of 34 interventions found that only one-third provided some form of educational information about how to substitute added sugars with alternatives [20]. Other added sugar studies have included non-animated, web-based videos, such as a puppet show [21], an expert opinion intercut with case studies [22], video courses [23], and storytelling interviews [24]. These interventions ranged from approximately 6-15 minutes in duration, which is longer than the optimal time required for a social media format [21-24]. In addition, the interventions were specific to demographic groups (eg, Filipinos, Taiwanese, Somalis, and Latinos) and populations (eg, children, patients with type 2 diabetes, mothers, and cultural minorities) [21-24]. One recent study reports that a non-narrative based health message produced significantly higher behavioral intent to limit sugar intake than the narrative based comparison [25], while other studies have reported positive changes in health-related behaviors after exposure to narrative-based video interventions [9,26,27].

To evaluate the effectiveness of our intervention, we used a randomized controlled trial (RCT) to assign participants to a SAS video about reducing added sugar intake, or a SAS video about sunscreen use (the content placebo), or a SAS video about earthquakes (the placebo). We hypothesize that the exposure to the intervention video will improve behavioral intent to reduce the consumption of added sugars when compared to the content placebo and placebo videos. Intervention effectiveness will be measured by higher behavioral intent scores in the sugar intervention arm. We also hypothesize that the exposure to the intervention video will attenuate the direct restoration of freedom to consume added sugars when compared to the content placebo and placebo videos. No statistically significant difference in the direct restoration scores between the intervention and placebo videos will indicate intervention effectiveness. The findings from this study will provide insights for the understanding of reactance-related processes and their effect on behavioral intent in health promotion.

## METHODS

### Trial design

In this web-based RCT, we randomized participants 1:1:1 to a SAS video about added sugars (the intervention arm), a SAS video about sunscreen use (the content placebo arm), and a SAS video about earthquakes (the placebo arm). We nested six list experiments in each trial arm. For each list experiment, we randomized participants 1:1 to a control or treatment group. We motivate our use of the content placebo and placebo arms as well as the list experiment in the *Procedures* section.

### Trial registration

This study was registered at the German Clinical Trials Register (www.drks.de) on July 24^th^, 2020: DRKS00022340.

### Participants

We used the Prolific platform to recruit the web-based study participants. Inclusion criteria included being between the ages of 18 and 59 years (male, female, or other), being able to speak English, and having residence in the United Kingdom (UK). Exclusion criteria were not any of the inclusion criteria. We recruited participants on a ‘first come, first served’ basis until the recruitment number (sample size) was reached. Participants were provided with an informed consent form on the Prolific platform, which explained the purpose of the study, the risks and benefits of the research, and a means by which a participant could contact the researcher or the human subjects review board at Heidelberg University. After consenting, Prolific redirected participants to the Gorilla platform, where the study was hosted. Gorilla is a cloud platform that provides versatile tools to undertake web-based, experimental, and behavioral research. We paid participants £1 (US$1.38) for the 10-minute completion time.

### Procedures

Participants were asked basic demographic questions about their age, gender, and highest education completed. The Gorilla algorithm then randomly assigned participants to the trial arms. Participants watched one SAS video once from start to finish.

The sugar intervention video is animated, completely in English, and 3.42 minutes long. It was developed by our co-author (MA) at the Stanford School of Medicine and designed for a diverse and global audience. The two main characters, a mother and her pre-adolescent daughter, engage in food-related activities, such as grocery shopping and cooking dinner. The sugar video presents educational content on health problems that arise from consuming added sugars in foods, such as yoghurt, chocolate milk, and breakfast cereals. The narrative also includes the story of the father in this family who dies from diabetes-associated complications from the frequent consumption of soda drinks. It concludes with a text message from the World Health Organization (WHO) about the recommended maximum number of teaspoons of sugar per day.

The content placebo video is similar in style to the sugar video: it is animated, has a length of 3.42 minutes, and promotes a health message about the use of sunscreen [28]. We use the content placebo video to isolate the *content effect* of the sugar intervention video as it does not include the content about added sugars. The placebo video about earthquakes [29] is also animated and has the same length as the sugar and sunscreen videos. It describes the causes and characteristics of earthquakes and contains no health-related message or added sugar message. Since the Sunscreen video promotes a health message and the Earthquake video does not, we attribute any significant difference in the end-points (following random assignment) to the content of the sunscreen message. We call this difference the *health awareness effect*. The *total intervention effect* is therefore the sum of the content and health awareness effects.

After random assignment, all participants provided responses to questions about threat to freedom (the antecedent to reactance), anger and negative cognition (the components of reactance), and source appraisal and attitude (the consequences of reactance), as shown in **Figure 1** and described elsewhere [30,31]. All reactance components were measured on a 5-point Likert scale: (1) strongly disagree; (2) disagree; (3) neither agree nor disagree; (4) agree; (5) strongly agree [32].

To reduce socially desirable responses to the behavioral intent and direct restoration questions, we nested six list experiments in each arm. **Table 1** shows the six list experiments and their items. We randomized participants to either a control or treatment group. The control group received a list of five items and the treatment group received the same list with one additional item. This item included the topic of natural vs added sugars, consumption of sugar-sweetened beverages, fresh fruit intake, reading of sugar content on package labels, teaspoons of sugar consumed per day, and home cooking. Each list experiment was preceded by the question: “How many of the five/six statements do you agree with? We don’t want to know which ones, just answer how many.” The justification and methodology for the list experiment is described in greater detail elsewhere [30,33].

**Table 1:** The six list experiments*

List 1: Added vs natural sugar	List 2: Sugar-sweetened beverages
This week I feel *motivated* to ...	This week I feel *unmotivated* to ...
1. Spend time watching TV	1. Wash my hands frequently
2. Do the vacuuming in my home	2. Spend time watching movies
3. Spend time chatting with my friends online	3. Clean the toilets in my home
4. Pick a fight with my partner	4. Smoke marijuana
5. Rinse my nose with salt water daily	5. Clip my toenails
6. Cut my daily intake of added sugar [in the treatment list only]	6. Reduce the amount of sugar-sweetened beverages I drink [in the treatment list only]
**List 3: Fresh fruit**	**List 4: Food labels**
This week I feel *motivated* to...	This week I feel *unmotivated* to...
1. Open up a new savings plan at the bank	1. Clean my dishes after use
2. Practice playing a musical instrument	2. Spend time on the internet
3. Watch a pornographic movie	3. Try learning a new language
4. Do some online shopping	4. Play a prank on my partner
5. Clean kitchen counters after use	5. Visit an online car sales website
6. Eat fresh fruit daily [in the treatment list only]	6. Count how many teaspoons of added sugar I eat each day [in the treatment list only]
**List 5: Teaspoons of sugar**	**List 6: Home cooking**
This week I feel *motivated* to...	This week I feel *unmotivated* to...
1. Watch a new TV series	1. Stock up on household supplies for a month
2. Practice meditation daily	2. Spend time gardening by myself
3. Have alcoholic drinks on at least three evenings	3. Plan my next holiday
4. Catch up on last week’s work	4. Take an online course
5. Clean all floor surfaces	5. Go out with my friends
6. Check food labels for sugar content [in the treatment list only]	6. Cook with fresh, whole foods [in the treatment list only]

*In each trial arm, participants were randomized (1:1) to a control or treatment group. Both groups received six lists. For each list, the control group received the first five items only; the treatment group received the five items and a sixth item. The items in each list were randomly ordered. Each list experiment was preceded by the question: “How many of the five/six statements do you agree with? We don’t want to know which ones, just answer how many. This week I feel motivated (List 1, 3, 5) or unmotivated (List 2, 4, 6) to ...”.

### Outcome measures

We assessed two outcomes: the mean difference in 1) behavioral intent to reduce the consumption of added sugars and 2) direct restoration of freedom following exposure to the intervention or placebo message. To assess behavioral intent, we used Lists 1, 3, and 5, which begin the 5/6 items with the words “This week I feel *motivated …*”. To assess direct restoration, we used Lists 2, 4, and 6, which begin the 5/6 items with the words “This week I feel *unmotivated …*”. To avoid order effects, we made Gorilla present the six list experiments in a random order, with the items in each list presented in random order. We constructed the items to minimize ceiling and floor effects.

### Statistical methods

We used descriptive statistics to obtain means and standard deviations of gender, age, and education status. For the primary endpoints we calculated the prevalence of behavioral intent to reduce added sugar intake using Lists 1, 3, and 5. We counted the mean number of items that participants selected from the control list (min = 0, max = 5) and the mean number of items that the participants selected from the treatment list (min = 0, max = 6). For each list experiment, we then estimated the prevalence of behavioral intent, defined as the difference in the mean behavioral intent scores between the treatment and control group [34]. From these estimates, we calculated the content effect as the difference in mean scores on a scale of 0-100 between the sugar and sunscreen arms. We also calculated the total intervention effect as the difference in mean scores on a scale of 0-100 between the sugar and earthquake arms. We obtained these estimates by specifying the main and interaction terms in an ordinary least squares (OLS) regression model, which is similar to a difference-in-difference analysis. We calculated standard errors, 95% confidence intervals, and *P* values for linear combinations of coefficients from the OLS model. For hypothesis testing for the difference in means, we used a t-distribution and set a significance level at 0.05. Using regression models specifically designed for list experiments, we modelled the effect of each reactance component on behavioral intent [34]. We used all of the above procedures for the direct restoration experiments (List 2, 4, 6). Notation for the end-point calculations can be found in the Supplement and the sample size calculation has been described elsewhere [30]. All statistical analyses were performed using the statistical software R (version 4.0) (Foundation for Statistical Computing, Vienna, Austria).

### Protocol

The full protocol has been published and can be accessed at any time [30].

#### Availability of data and materials

Data has been collected and stored on the Gorilla platform. The study investigators own and have complete control of the research data, which can be accessed at any time. For statistical analysis, the data was downloaded and is safely stored on a computing system maintained by the Heidelberg University.

### Blinding

The participants were completely anonymous to the study investigators as the Prolific platform handled the interaction between the study investigators and participants. Only the participant’s unique, anonymized ID was used to manage the linking between the Prolific and Gorilla platforms. The outcome measures were self-reported and submitted anonymously. The study investigators and those involved in the data analyses were blinded to the group allocation.

### Adverse event reporting and harms

Because the participants were anonymous to us, we were not able to report any adverse events or harms. It is unlikely there were any adverse events given the format of our 10-minute web-based trial.

## RESULTS

**Figure 2:**
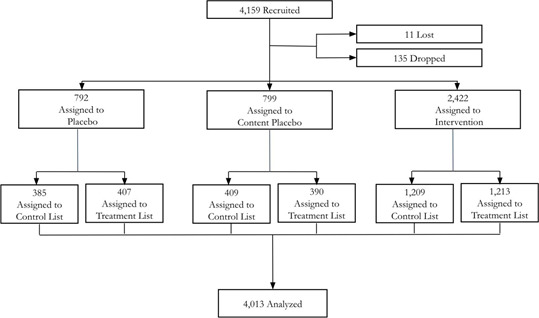
Trial design.

We recruited 4159 participants from the UK on the 9^th^ and 10^th^ December 2020. After enrollment, 146 (3.5%) participants did not finish the trial either due to technical reasons (poor internet connection, video loading issues, system crash, etc.) or other unknown reasons. In total, 4013 participants (96.4% of the recruited sample) completed the trial and were included in the final analysis (**Figure 2****).** We present the baseline characteristics of the participants by trial arm, including gender, age, and highest education level in **Table 2****.** Of the final sample, 60.9% were female, 32.3% were between 25-34 years of age, and 64.1% had some college education or a Bachelor’s degree. We observed no statistically significant differences in baseline characteristics across the three trial arms.

**Table 2:** Summary of demographic characteristics by trial arm (N = 4013)*

	Placebo (earthquake video), n = 792		Content placebo (sunscreen video), n = 799	Intervention (sugar video), n = 2422	*P*-value
**Gender:**					0.486
Female		485 (61.2%)	481 (60.2%)	1476 (60.9%)	
Male		300 (37.9%)	313 (39.2%)	937 (38.7%)	
Other		7 (0.88%)	5 (0.63%)	9 (0.37%)	
**Age (year):**					0.882
18-24		208 (26.3%)	184 (23.0%)	609 (25.1%)	
25-34		250 (31.6%)	259 (32.4%)	787 (32.5%)	
35-44		167 (21.1%)	175 (21.9%)	532 (22.0%)	
45-54		120 (15.2%)	130 (16.3%)	350 (14.5%)	
55-59		47 (5.93%)	51 (6.38%)	144 (5.95%)	
**Education:**					0.783
Primary school or less		11 (1.39%)	13 (1.63%)	27 (1.11%)	
Completed high school		126 (15.9%)	123 (15.4%)	374 (15.4%)	
Some college, BA		500 (63.1%)	501 (62.7%)	1573 (64.9%)	
MA, PhD		155 (19.6%)	162 (20.3%)	448 (18.5%)	

*Data are presented as number (n) and proportion (%). We used the χ2 test to compare the demographic variables in three trial arms. A *P*-value <0.05 was considered statistically significant. The *P*-values indicate that there were no significant differences in age, gender, or educational status between the trial arms.

The mean scores for the six list experiments by trial arm and list treatment group are shown in Figure S1 in the **Online Supplementary Document**. The difference in mean scores between the treatment and control groups gives the prevalence of behavioral intent (Lists 1, 3, 5) and intent to restore freedom (Lists 2, 4, 6) toward added sugar intake. Scores in the treatment group (6 items) are higher than in the control group (5 items) because of one additional item. These differences represent the prevalence toward behavioral intent or direct restoration of freedom. For example, in List 1 (of Figure S1 in the **Online Supplementary Document**), the mean score for the intervention arm is 2.7 for the treatment group and 2.31 for the control group. Therefore, the prevalence of behavioral intent to cut the daily intake of added sugar is 39% (2.7-2.31 × 100).

**Figure 3:**
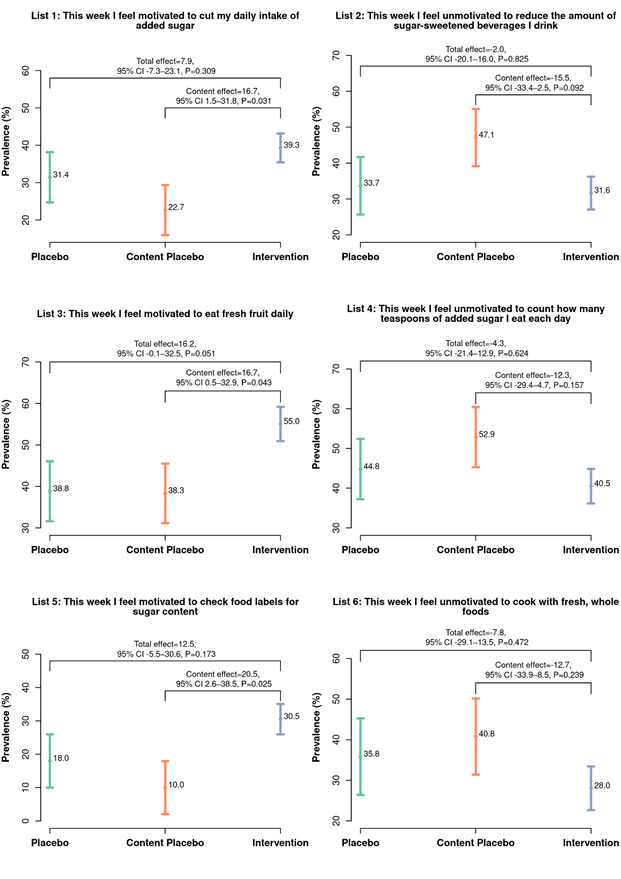
Prevalence of behavioral intent (Lists 1, 3, 5) and direct restoration (Lists 2, 4, 6) with 95% CIs by trial arm. The results show that the prevalence of behavioral intent to reduce added sugars was significantly higher among participants who watched the sugar intervention video, compared to participants in the two placebo arms (Lists 1, 3, 5). We also show that the sugar intervention video aroused lower motivation to restore freedom when compared with the two placebo videos (Lists 2, 4, 6), although these differences were not statistically significant (which is further evidence in support of the effectiveness of the intervention).

In **Figure 3****,** we compare behavioral intent (List 1, 3, 5) to reduce added sugars (after removing social desirability bias) between the sugar, sunscreen, and earthquake videos. Results show that participants who watched the sugar video had significantly higher behavioral intent to cut their daily intake of added sugar (16.7, 95% CI = 1.5-31.8, *P* = 0.031), eat fresh fruit daily (16.7, 95% CI = 0.5-32.9, *P* = 0.043), and check food labels for sugar content (20.5, 95% CI = 2.6-38.5, *P* = 0.025) than those in the content placebo arm. We also observed high behavioral intent to reduce the intake of added sugars between the sugar and earthquake videos but these differences were not statistically different. In **Figure 3**, we compare behavioral intent to restore freedom (Lists 2, 4, 6) between the sugar, sunscreen, and earthquake videos. We show that there were no statistically significant differences between the three videos, which implies that the sugar video did not arouse reactance to the sugar message.

**Figure 4:**
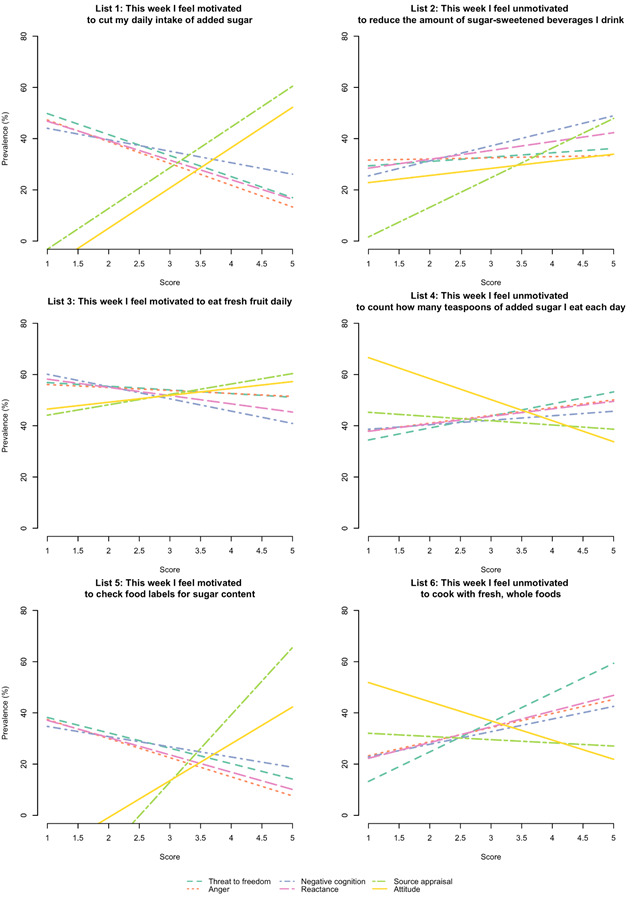
Relationship between the reactance components and behavioral intent (Lists 1, 3, 5) and direct restoration (Lists 2, 4, 6) for the six list experiments. In List 1, for example, a positive source appraisal of the narrator and a favourable attitude toward the sugar message is associated with higher motivation (behavioral intent) to cut the daily intake of sugar. In List 4, an unfavourable attitude toward the sugar message is associated with higher scores for unmotivated intent to count the amount of teaspoons of added sugar (direct restoration).

For each list experiment, we also assessed the effect of the six reactance components on behavioral intent and direct restoration. More detailed results for the antecedents, components, and consequences of reactance are presented elsewhere [31]. The plots in **Figure 4** show how behavioral intent (Lists 1, 3, 5) and direct restoration (Lists 2, 4, 6) change for a 1 unit increase in each reactance component. Results show that participants who had a higher source appraisal score (eg, ‘The narrator of this video was knowledgeable’) and a more favourable attitude score (eg, ‘I agree with what the message recommends’) toward the sugar message were more likely to have higher behavioral intent to reduce their consumption of added sugars. Participants who perceived the message to be threatening (eg, ‘The message threatened my freedom to choose’), or who were angered by the message (eg, ‘This message makes me feel angry’) or had negative cognition (eg, ‘The thoughts I had while watching this video were mostly negative’), or reactance (both anger and negative cognition) were more unmotivated to reduce their consumption of added sugars. Thus, participants with higher scores on these four components were more likely to reject the sugar message. In addition, as the source appraisal and attitude scores decreased, the intent to restore freedom increased (except for List 2 in **Figure 4****).**

## DISCUSSION

Short and animated (SAS) videos present a promising and effective strategy for promoting healthy behaviors. Such videos can engage large and diverse audiences in evidence-based health education because they use a story format, are emotionally compelling, and can be rapidly distributed through social media channels. Since health education is a cornerstone for changing behaviors, it is crucial to improve the design of SAS videos to increase the long-term impact of such interventions. In this study, we assessed the effectiveness of a SAS video to improve behavioral intent to reduce the consumption of added sugars and to attenuate the direct restoration of freedom to consume added sugars.

Our results show that participants who watched the sugar video had significantly higher behavioral intent to reduce added sugars when compared with those who watched the sunscreen (content placebo) video. Specifically, participants who watched the sugar video were more motivated to cut their daily intake of added sugar, eat fresh fruit daily, and check food labels for sugar content. For these three sugar-related behaviors, behavioral intent was approximately 16% higher than the sunscreen video and 12% higher than the earthquake video. Based on these results, we find evidence in favour of our first hypothesis, which states that exposure to the sugar intervention will improve behavioral intent to reduce the consumption of added sugars when compared to the placebo videos.

In this study, we also investigated if participants were likely to reject the sugar message. We did this by measuring the participant’s attempt to restore their freedom. The theory of reactance posits that individuals will seek to restore their freedom after it has been threatened with elimination by the health message. Our results show that participants who watched the sugar video did not arouse intent to restore freedom significantly more or less than the placebo videos. Therefore, we cannot support our second hypothesis.

An important contribution of our study is that we modelled the effect of the reactance components on behavioral intent and direct restoration outcomes. This was made possible by recent advances in list regression methods [34]. Our analysis shows that positive appraisal of the source and a favorable attitude towards the sugar message resulted in higher behavioral intent and lower intent to restore freedom. On the other hand, participants who initially scored higher on the ‘negative’ components of the reactance (threat to freedom, anger, negative cognition) were less motivated to change their behavior and more willing to restore their freedom. The results of our study show that health messages that threaten one’s freedom and arouse reactance will be less successful at nudging a person toward the targeted health behavior.

Our study has several strengths. First, we used an RCT design, which is the gold standard for evaluating the causal impact of an intervention on a health behavior outcome. Here, we randomized participants to either the sugar video intervention, a content placebo (the sunscreen video) or a placebo (the earthquake video). The content placebo is an innovative feature of our study, which enabled us to quantify the content effect as the difference in mean behavioral intent between the sugar video (the intervention) and the sunscreen video (the content placebo). Since both videos promote a health message, and because of randomization, any significant difference in mean scores should be due to the sugar reduction content of the intervention video. Similarly, we calculated the total intervention effect as the difference in mean scores between the sugar video and the earthquake video (the placebo). We are not aware of any previous study that has used an experimental approach to partition the effect of a SAS intervention video in this way. A third strength is that we used list experiments to indirectly obtain truthful responses and reduce social desirability bias. Here, we assumed that most participants know that consuming added sugars is unhealthy and that they will give socially desirable responses. By asking participants to count their agreement with six items, we could indirectly estimate the prevalence of participants that truly intend (in the next week) to reduce their consumption of added sugars.

In a previous study using the same data [31], we evaluated the effect of social authority on psychological reactance to the same sugar video described in this study. We randomized participants to the same placebo videos (sunscreen and earthquake) or the same sugar intervention video narrated by either a child (low social authority), the child’s mother (equivalent social authority), or the family doctor (high social authority). Our hypothesis was that a child narrator would arouse less reactance to the sugar message, because such a narrator would be perceived as less threatening or lacking an ‘ulterior motive’. We found no significant differences in reactance component scores between the three sugar narrators (child, mother, and doctor) [31]. For this study, we therefore treated the three narrated intervention videos as a single intervention video, thus explaining the higher number of participants randomized to the sugar video compared to the placebo videos.

Our study has several limitations. One limitation is that we financially compensated participants, which was unavoidable given the web-based format of our study. Because of financial compensation, some studies may attract economically disadvantaged participants, who will be disproportionately represented and may have less individual autonomy when given the choice for a lucrative incentive [35]. In our study, participants were paid £1 (US$1.38) for an expected 10-minute completion time, which is fair (and close to the UK minimum wage of £6 (US$8.28) per hour for 18-20 year-olds) but not very lucrative [36]. Furthermore, given that 63.6% of the participants had a Bachelor’s degree (or some college) and 83.2% had a Bachelor’s degree or higher (MA, PhD), it is unlikely that our study disproportionately attracted vulnerable or economically disadvantaged participants. It is also widely accepted to compensate healthy participants when the research is not focused on a specific disease or treatment and does not involve potential risks [37]. Another limitation is that we were only able to assess short-term behavioral intent to reduce the consumption of added sugars. The impact of our sugar intervention on long-term behavioral change remains unknown. However, our results show a promising impact on short-term behavioral intent, which is a critical first step toward adopting a new behavior [38]. Furthermore, we also demonstrated that the sugar intervention aroused less (but not statistically significant) reactance when compared with the two placebo videos, which is a positive finding. This finding suggests that SAS videos can be an important component in the value chain of promoting health behaviors.

## CONCLUSIONS

Overall, the findings of this study demonstrate that our SAS intervention can successfully attenuate reactance and improve short-term behavioral intent to reduce the consumption of added sugars. Importantly, our SAS video did not arouse the participants’ motivation to restore their freedom, indicating the positive impact of the video on the behavioral change. In addition, the results of this study confirm that positive source appraisal and attitude towards the message can serve as reliable predictors of higher behavioral intent and lower freedom restoration, whereas greater threat to freedom and reactance (composed of anger and negative cognition) may result in lower behavioral intent and higher freedom restoration. These findings can be further used to inform the design of SAS videos and are aligned with existing evidence about the use of powerful video-based messages in health communication [4,6,39]. This work contributes to a larger body of research investigating innovative approaches to digital health messaging that leverage best practices from communication theory, entertainment media, and health education. The goal of such innovation is to meet our audiences where they are (on social media) and to convey science-based health messages that are accessible and engaging across a broad spectrum of cultures, languages, ages, and educational backgrounds.

## Additional material


Online Supplementary Document
