# Supplementary material for: Effect of a story-based, animated video to reduce added sugar consumption: A web-based randomized controlled trial
Source: J Glob Health. 2021 Oct 30;11:04064. doi: 10.7189/jogh.11.04064 (PMC8564880; doi:10.7189/jogh.11.04064)
Supplement: Online Supplementary Document [file jogh-11-04064-s001.pdf]

Supplement

We analyzed list experiments to measure prevalence of behavioral intent and restoration of threatened freedom. Let C_j denote the number of items that the jth participant selected from the controllist (min. = 0, max. = 5), and let T_j be the number of items that the jth participant selected from the treatment list (min. = 0, max. = 6). The means score was calculated for the control list, denoted by $\bar{C}_i$, and treatment list, denoted by $\bar{T}_i$, for the ith list experiment (i = 1, . . . , 6). Let the superscripts ^{i} denote the Intervention arm, ^{cp} denote the content placebo arm, and ^{p} denote the placebo arm, and let k denote the kth trial arm ($k \in$ [i,cp,p]). For each list experiment i and trial arm k, we then estimated the prevalence of behavioral intent and restoration of freedom, denotedby P^k, as the difference between the treatment and control, such that $P^k = (\bar{T}^k - \bar{C}^k) \times 100$. From these estimates, we calculated the total and content effects of the intervention video. Let D^{Tot} denote the total effect, which is estimated by $P^i - P^p$ and let D^{Cont} denote the content effect, which is estimated by $P^i - P^{cp}$. These analyses are analogous to difference-in-difference analyses, which we implemented by specifying the main and interaction terms in an ordinary least squares(OLS) regression model. The OLS equation for the ith list experiment is given as:

$$y = b_0 + b_1\text{VideoArm} + b_2\text{TreatList} + b_3(\text{VideoArm} \times \text{TreatList}), \qquad (1)$$

where y is the number of statements in the list that the participant agreed with, VideoArm indicates the kth arm, and TreatList indicates assignment to the treatment or control list.

Sample size

We calculated the sample size needed for pairwise comparisons between three groups using a one-way analysis of variance (ANOVA). The formula to calculate the sample size is [1]:

$$n_A = (\sigma_A^2 + \sigma_B^2/\kappa)\left(\frac{z_{1-\alpha/\tau} + Z_{1-\beta}}{\mu_A - \mu_B}\right)^2 \tag{2}$$

where $\kappa = 1$, which is the matching ratio, μ_A and μ_B are the group A and B means, σ_A and σ_B are the group A and B standard deviations, $\alpha = 0.05$ is the Type-I error, $\beta = 0.20$ is the Type-II error, z is the quantile function, and $\tau = 2$ is the number of comparisons to be made. We assumed a mean of $\mu_A = 2.0$ and $\mu_B = 2.15$ for the control and treatment groups, respectively. In other words, we expect, on average, that the control group will agree with 2 out of the 5 items and the treatment group with 2.15 of the 6 items. We selected $\sigma_A = 0.85$ and $\sigma = 1.0$. This calculation gives a sample size of $n = 769$ per group. For the 5-way comparison, the sample size is $n = 3{,}845$. To ensure we have sufficient power and account for attrition, we selected a sample size of $n = 4{,}000$. In this study, we did a 3-way comparison between the sugar intervention and the two placebo groups. Since the number of comparison groups decreased, the n for this study is sufficient.

REFERENCES

1. Rosner B. Fundamentals of biostatistics. 7th ed. Boston, MA: Cengage Publishers; 2010. ISBN:29005387733495

Supplement figures

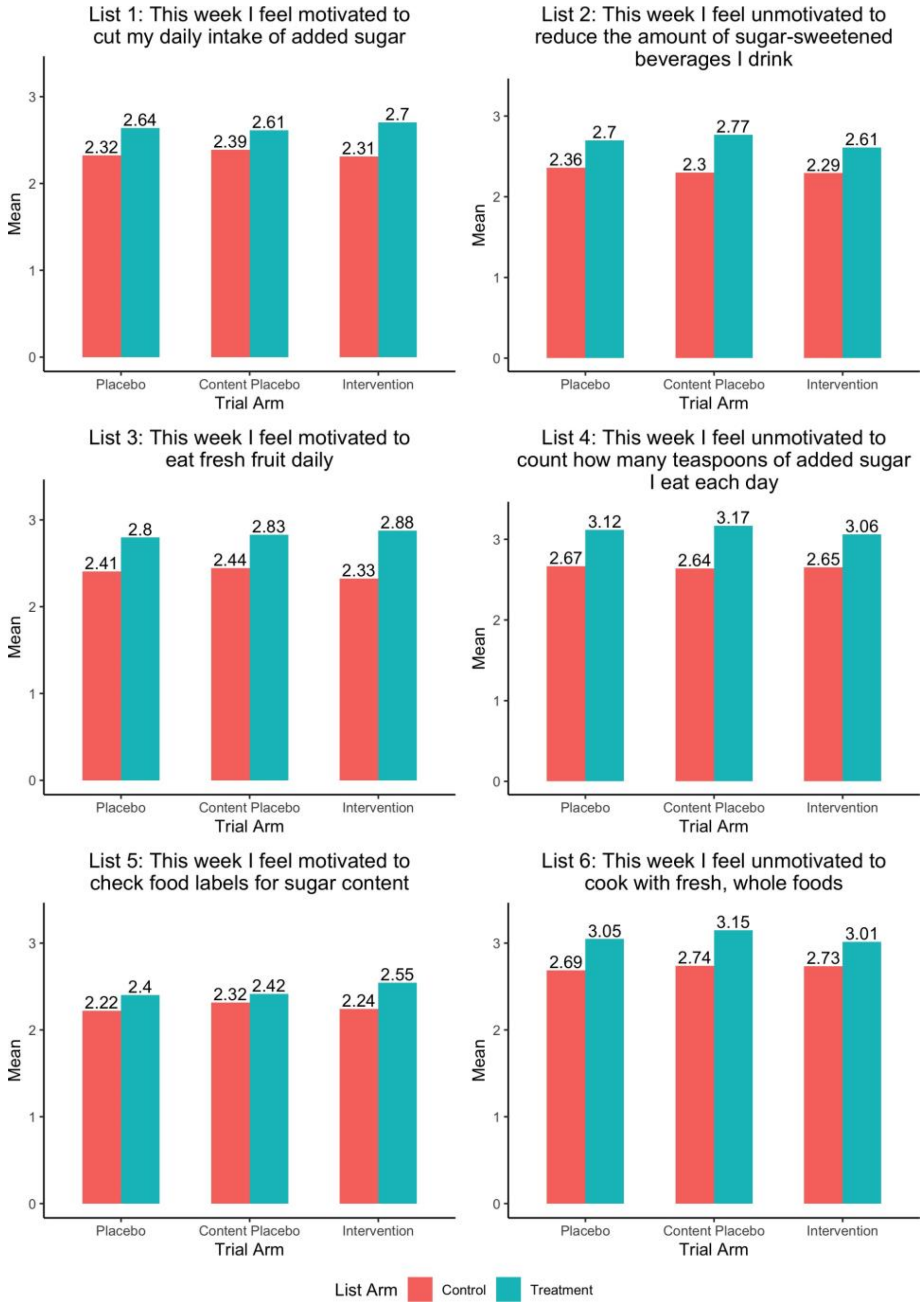

Figure S1: Mean scores for the 6 list experiments by intervention (sugar video), content placebo (sunscreen video), and placebo (earthquake video) arm and by list arm (treatment list and control list). The mean scores represent the mean number of items that participants selected from the control list (5 items) or treatment list (6 items). Mean scores in the treatment list will always be higher because this list has an additional ‘sensitive‘ item about added sugars. The difference in treatment and control list means gives the prevalence of participants that were motivated (Lists 1, 3, 5) or unmotivated (List 2, 4, 6) to reduce added sugars. For example, in List 1, participants randomized to the treatment list had a mean score of 2.7 versus 2.31 for the control list. Thus, the prevalence of participants motivated to cut their daily intake of sugar is 39%.
